# Identifying Geographic Inequities in Family Planning Service Uptake in Pakistan: A Comparative Study of PDHS 2006 and 2017 Using Cluster Hotspot Analysis

**DOI:** 10.3390/women4040028

**Published:** 2024-10-10

**Authors:** Kamran Baig, Ebele Okoye, Mary Shaw

**Affiliations:** 1 Sindh Social Protection Authority, Karachi 75500, Pakistan; 2 Department of Epidemiology & Biostatistics, College of Health Sciences, Jackson, MS 39213, USA; 3 Department of Behavioral & Environmental Health, College of Health Sciences, Jackson, MS 39213, USA

**Keywords:** contraception, family planning, gender inequity, hotspot analysis, maternal health, Pakistan

## Abstract

Family planning (FP) services are crucial interventions for improving maternal and child health outcomes and promoting gender equity. However, ensuring equitable access to these services remains a significant challenge, particularly in countries like Pakistan, where sociocultural norms, economic disparities, and geographic barriers hinder FP uptake. This study utilized spatial analysis techniques, including hotspot analysis, to investigate geographic disparities in FP uptake in Pakistan using data from Pakistan Demographic and Health Surveys (PDHS) conducted in 2006–2007 and 2017–2018. ArcMap 10.1 was used for spatial analysis and Stata 12.0 for statistical analysis. Results revealed significant spatial variations in FP uptake, with urban areas exhibiting higher uptake rates than rural regions. Hotspot analysis identified dynamic changes in contraceptive prevalence rates (CPR), with significant clustering in some regions and dispersion in others. It also identified areas with high unmet need, low intention to use FP services, and preference for family size (>3 children), highlighting the need for targeted behavioral change interventions. This innovative spatial approach provides nuanced insights for policymakers and program planners to develop targeted interventions based on localized data to improve FP service delivery, mitigate disparities, and ultimately advance efforts to improve maternal and child health outcomes. The application of geospatial analysis is an effective tool for enhancing program planning, evaluation, and resource allocation in diverse geographical contexts.

## 1. Introduction

Family planning (FP) has the potential to save over 30% of maternal and 10% of newborn deaths worldwide, making it one of the most important public health concerns and one of the most “health-promoting” and economical public health promotion initiatives [1]. It includes all the goods, services, information, commodities, attitudes, and policies—including contraceptives—that encourage couples to choose when and whether to have a child and prevent unintended pregnancies [2].

Enhancing the accessibility of family planning services improves maternal and child health and promotes gender parity and women’s empowerment [2]. Despite these benefits, equal access to FP services is a challenge in Pakistan because of a lack of education, poor knowledge of FP, and low socioeconomic status, ultimately affecting the utilization of FP services in Pakistan [3]. Several other health system-related factors, such as inadequate health service delivery, poor outreach coverage, and ineffective FP programs, have been linked with the under-utilization of FP services in Pakistan [4,5].

Pakistan is experiencing exponential population growth, which poses significant challenges; hence, there is a need for enhanced uptake and continuation of FP services. Globally, Pakistan is rated the fifth most populated nation, with about 225 million people. However, according to estimations by the Pakistan Bureau of Statistics (PBS) and the United Nation’s World Population Prospects (WPP), the population will grow to 310 million by 2050 [6,7]. In 2012, the Pakistani government promised to commit its efforts to address this challenge by increasing the nation’s contraceptive prevalence rate (CPR) to 55% by 2020, which was then changed to 50% by 2025 [8]. However, since 2007, Pakistan’s CPR has been stagnant between 30–35% despite political efforts, allotted funds, and extensive FP programs [9]. Variation still occurs in the usage of FP services in different regions of Pakistan, especially in the rural areas. Although the government and several stakeholders in the FP domain have endeavored to promote FP, challenges still exist in increasing the uptake and continuation of contraceptives. Pakistan faces difficulties in attaining sustainable development, lowering population growth, and enhancing maternal health [10].

Notably, Pakistan’s geographic areas show wide variations in CPR. The 2017 and 2018 PDHS reported that modern CPR was the highest (35%) in Islamabad and the lowest (14%) in Balochistan [11]. These inequities were relatively higher at the district level, as indicated by the Multiple Indicator Cluster Survey (MICS) [12]. This issue underlines the need for targeted or tailored approaches/interventions to reach the most marginalized and vulnerable women in Pakistan [13]. However, despite the continuous efforts of the government, many potential barriers still exist to the use of contraceptives, especially among women of childbearing age in Pakistan, because of the social, cultural, and perceived religious unacceptability of contraception, lack of knowledge and awareness of contraception, cost of contraception, and access to contraceptive services [3,14,15].

Current research studies have highlighted the significance of spatial analysis techniques, like Cluster Hotspot Analysis and Geographic Information Systems (GIS), in identifying geographic inequities in family planning and reproductive health services uptake [16–19]. This study, however, aimed to add to the body of existing knowledge by investigating provincial or regional inequities in using FP services in Pakistan. It demonstrated the application of innovative techniques, such as geospatial analysis, in the evaluation and planning of family planning programs that can strategically transform the family planning landscape in Pakistan. It mapped geographic disparities in contraceptive prevalence rates, preferred family size, and intention to use FP services. The study underscores the critical importance of geospatial data and advocates for its integration into all future national and sub-national surveys and programs. This can assist policymakers, and program planners in identifying areas requiring immediate attention and resource allocation.

## 2. Materials and Methods

The study used the Pakistan Demographic and Health Survey (PDHS) collected in 2006–2007 and 2017–2018. The PDHS is a nationally representative household survey conducted every five years. The survey comprises two-stage cluster design sampling, which includes (a) clusters as primary sampling units that are drawn from census files and (b) secondary sampling units comprising 25–30 households that are randomly selected within each cluster. The DHS Program website (“Sampling and Household Listing Manual [DHSM4]”) provides further details on the sampling strategy. The DHS program collects GPS coordinates for the survey cluster or primary sampling unit. These coordinates were distorted to maintain the confidentiality and anonymity of the survey respondents. The clusters in rural areas were displaced 5 km from the center, and the clusters in urban areas were displaced 2 km from the center. Further details on the displacement can be accessed at the DHS program site [20].

### Statistical Analysis

Descriptive analysis was reported as frequency and percentage. The contraceptive prevalence rates were calculated using the master file provided by the DHS Program. The master file provides the “do” files to recode survey variables and perform tabulation to calculate prevalence rates [21]. All statistical analysis was done using Stata 12.0 (College Station, TX, USA), and spatial analysis was performed with ArcMap 10.1 (ESRI, Redlands, CA, USA). The study calculated the CPR for any method (aCPR), modern methods (mCPR), intent to use family planning services, and preference for an ideal family size for each cluster.

Moran’s I index was used to assess the autocorrelation in the clusters. It tests the null hypothesis in which values of aCPR, mCPR, and unmet need are randomly distributed. The index identified if there was any significant spatial pattern, clustered, dispersed, or random, in the data. It calculates Moran’s I index, including z-score and *p*-value, to evaluate the significance of the index. A statistically significant Moran’s I with *p* < 0.05 indicates rejection of the null hypothesis and the presence of spatial autocorrelation. The index value ranges from −1 to 1. A positive value indicates a clustered pattern, a negative indicates a dispersed pattern, and 0 shows a random distribution. A high z-score and a low *p*-value show a statistically significant deviation from a random pattern [22]. Hotspot analysis used the Getis-Ord Gi* statistics to identify statistically significant high values (hotspots) and low values (cold spots). The output feature class calculates z-scores, *p*-values, and confidence levels in fields. It is an inferential statistic that tests the null hypothesis that there is no spatial clustering of feature values. It is interpreted by analyzing the statistical significance of z-scores, *p*-values, and bin values of high and low clusters [23]. Interpolation was performed using the ordinary Kriging method that provides an optimum accuracy of predicted values for unsampled locations based on a weighted combination of nearby data points.

## 3. Results

There were 957 and 560 clusters in PDHS 2006–2007 and 2017–2018, respectively. The PDHS 2006–2007 had 385 (40.22%) clusters from urban areas and 572 (59.77%) clusters from rural areas, whereas the PDHS 2017–2018 had 282 (50.35%) clusters from urban areas and 278 (49.64%) clusters from rural areas. The spatial auto-correlation analysis indicated that the distribution of CPR of any method, CPR of the modern methods, and unmet need of family planning were not randomly distributed, as showed by the Global Moran’s I values in Table 1.

### 3.1. Hotspots Analysis of CPR for Any Method

**Figure 1.**
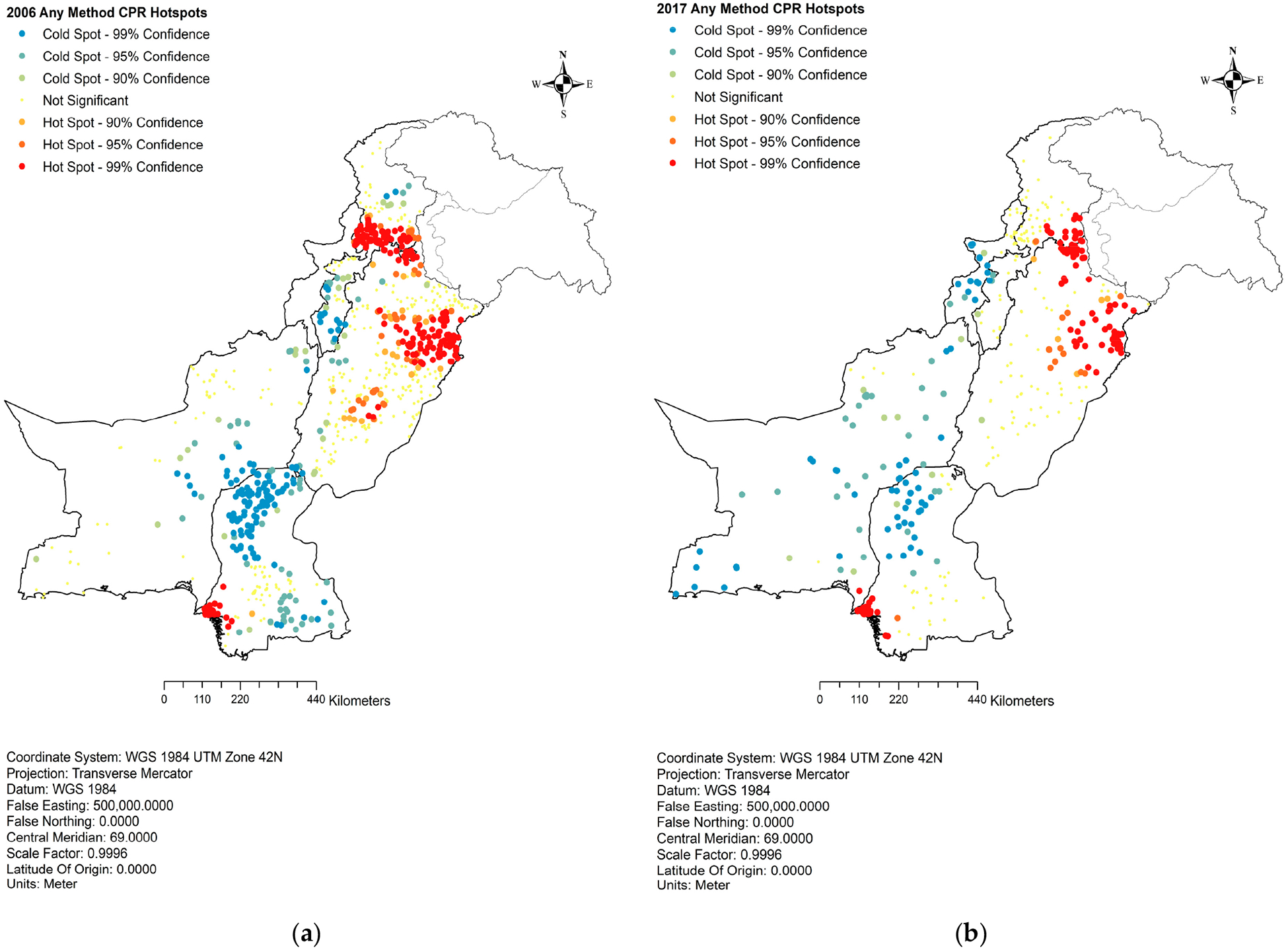
Hotspot analysis of contraceptive prevalence rate for any method. (**a**) Any method CPR hotspots, 2006; (**b**) any method CPR hotspots, 2017.

The hotspot analysis of contraceptive prevalence rate for any method (aCPR), based on the PDHS 2006–2007 data, revealed distinct spatial patterns, as indicated in Figure 1a. The areas with high aCPR were more clustered in lower or urban Sindh, upper or urban and certain parts of central Punjab, and the urban regions of Khyber Pakhtunkhwa (KP). Conversely, most Balochistan, Sindh, and KP exhibited the lowest CPR for any method during this period, as indicated by the significant cold spots.

Subsequent analysis of PDHS 2017 data observed a notable change in the spatial distribution of CPR for any method. The high CPR hotspots in Punjab had contracted and were primarily limited to the upper urban centers. In Sindh, the cold spots started diffusing but were still concentrated in the upper part, while the situation remained relatively unchanged in the other provinces, as presented in Figure 1b.

### 3.2. Hotspots Analysis of CPR for Modern Method

**Figure 2.**
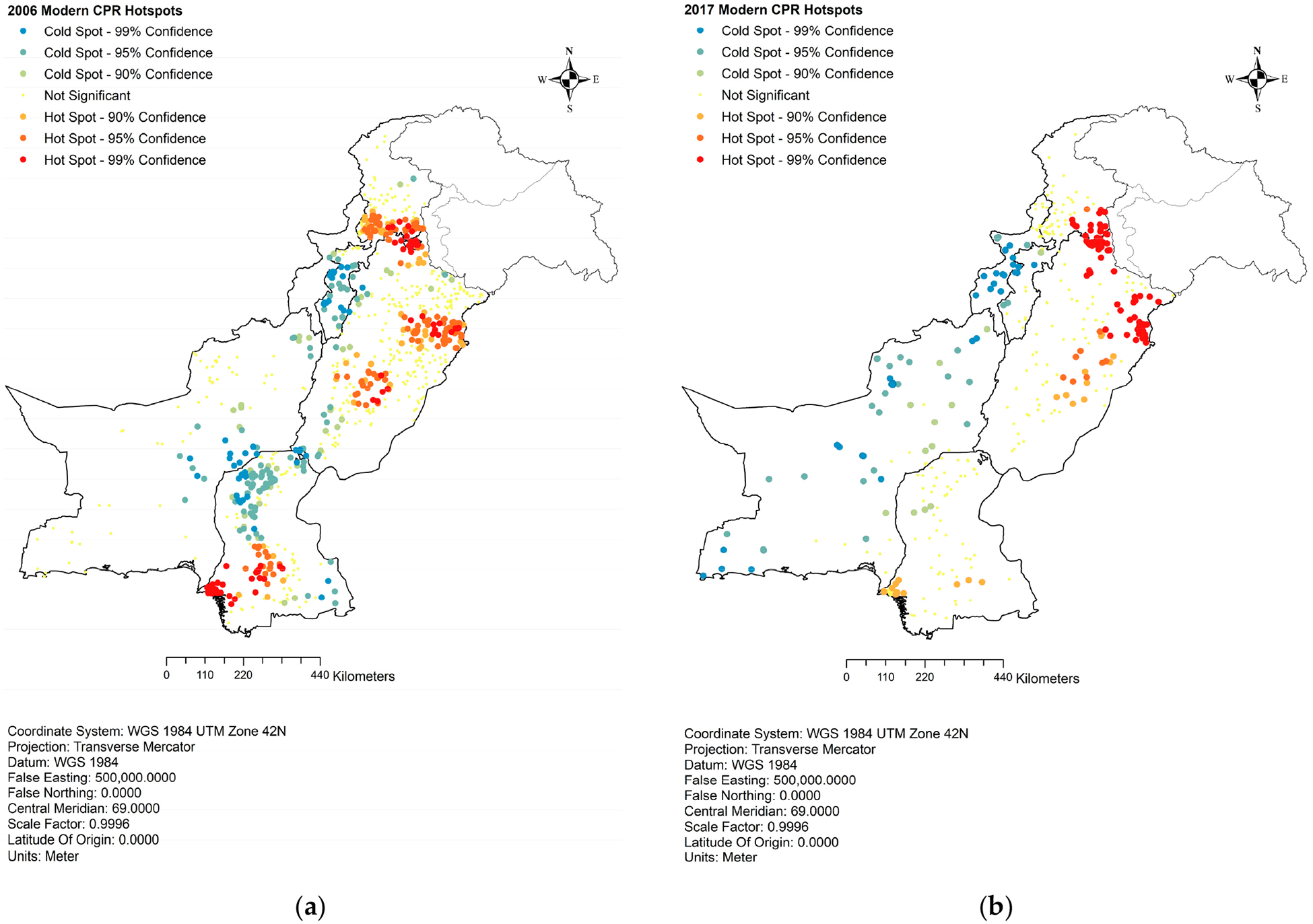
Hotspot analysis of contraceptive prevalence rate for modern method. (**a**) Modern CPR hotspots, 2006; (**b**) modern CPR hotspots, 2017.

The hotspot analysis of the contraceptive prevalence rate for the modern method (mCPR), based on the PDHS 2006–2007 data, revealed spatial patterns similar to the analysis of CPR for any method, as presented in Figure 2a. The areas with high mCPR were clustered in lower and central Sindh, upper and central Punjab, and urban areas of KP. Conversely, most of Balochistan, upper Sindh, and lower KP exhibited the lowest CPR for the modern method during this period. When analyzing the PDHS 2017 data, as indicated in Figure 2b, it was found that the most significant mCPR hotspots were identified in Punjab and had maintained almost a similar distribution as in 2006. In Sindh, the mCPR remained concentrated in the lower province, while central Sindh, a hotspot in 2006, did not maintain the same position in 2017, indicating a more uneven distribution of mCPR. Likewise, upper Sindh, which had a dense cluster of cold spots in 2006, did not show a similar pattern in 2017, and the clusters were diffused, indicating an improvement in the distribution of mCPR. The situation remained relatively unchanged in KP, but Balochistan exhibited some sporadic cold spots of mCPR as compared to 2006.

### 3.3. Unmet Need and Ideal Number of Children

**Figure 3.**
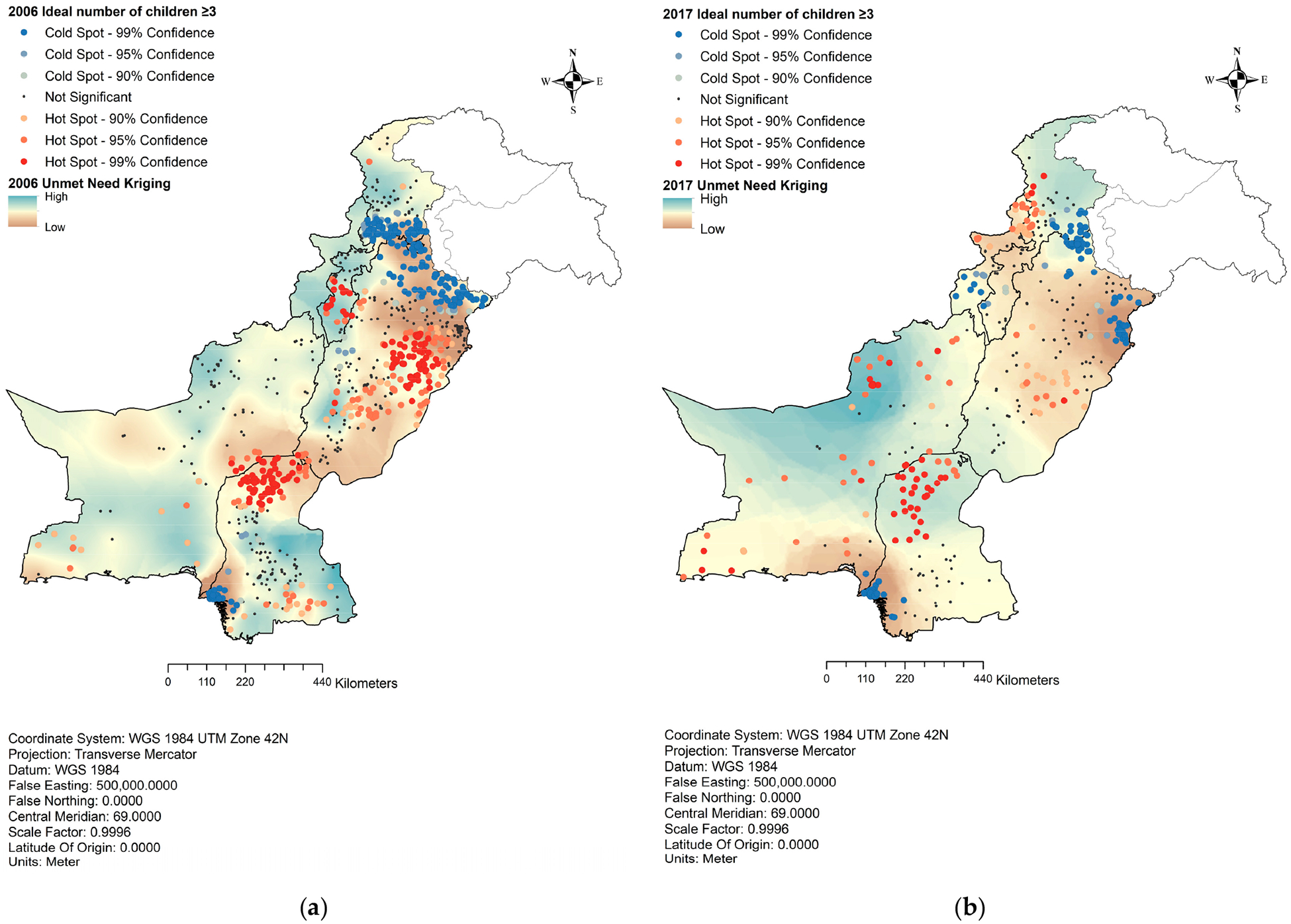
Hotspot analysis of unmet need and the ideal number of children. (**a**) Unmet need and preference for the ideal number of children in 2006; (**b**) unmet need and preference for the ideal number of children in 2017.

Our hotspot analysis of the ideal number of children with unmet need, presented in Figure 3, indicated that regions expressing a preference for three or more children were notably concentrated in areas with moderate to the highest unmet need. Metropolitan centers, which exhibited the lowest unmet need values, displayed cold spots for three or more children, indicating a prevalent inclination towards fewer (<3) children.

In 2006, the province of Sindh exhibited pronounced hotspot clusters of preference for more than three children in its northern part with low to moderate unmet need, accompanied by some sporadic clusters in the southern region with high unmet need. Similar trends were observed in Punjab, where significant hotspots of preferences for three or more children were marked in areas with moderate to low unmet need. In Khyber Pakhtunkhwa (KP), the hotspots were clustered in areas with high unmet need. Despite having the highest unmet need, Balochistan displayed only a few hotspots. The year 2017 showed notable shifts in spatial distribution, particularly in Balochistan and KP. Balochistan saw widespread hotspots throughout the province, primarily in areas with the highest unmet need.

Conversely, KP displayed cold spots for preferences of three or more children, even in areas characterized by moderate unmet need. In Sindh, the northern region with high unmet need remained a hotspot for clusters favoring three or more children. In central Punjab, sporadic hotspots emerged in the areas marked by moderate unmet need.

### 3.4. Unmet Need and Intention to Use Contrapcetives

**Figure 4.**
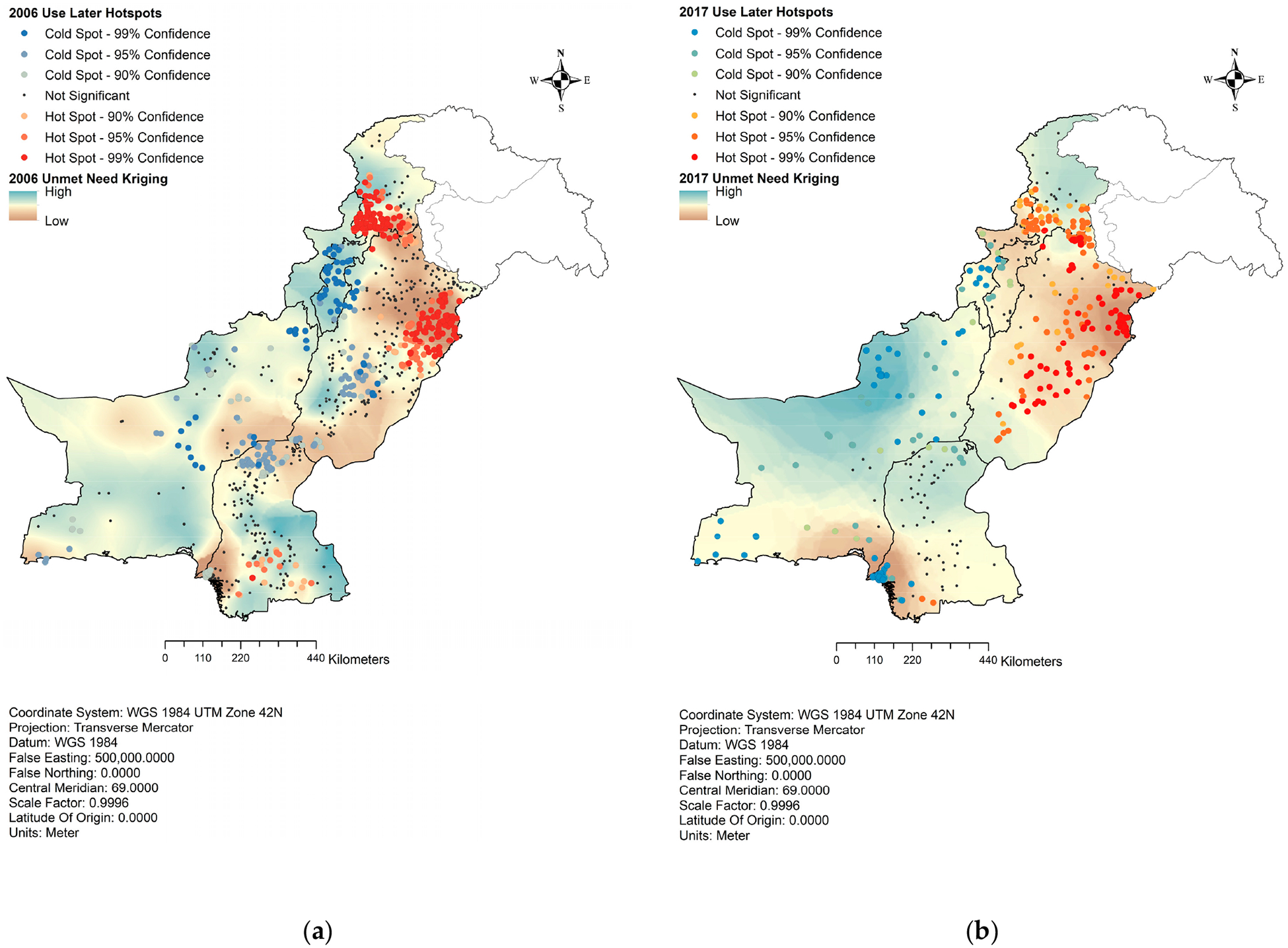
Hotspot analysis of unmet need and intent to use family planning methods later. (**a**) Use later hotspots, 2006; (**b**) use later hotspots, 2017.

This study assessed the spatial distribution of unmet need of family planning (FP) using the Kriging method. In 2006, statistically significant clusters indicating the intent to use FP services later were observed in areas of high unmet need in Sindh, as shown in Figure 4a. However, upper Sindh, which exhibited relatively low unmet need, had clusters with lower intention to use FP services later. In Punjab, the clusters intending to use FP services were more concentrated in areas with low unmet need. In contrast, central and lower Punjab demonstrated significantly lower proportions of intention to use FP services. In KP, the clusters with a high proportion of intent to use FP services were concentrated in areas with low unmet need. In contrast, despite having high unmet need, lower KP showed statistically significant cold spot clusters of intention to use them later. Balochistan had high unmet need in 2006 and sporadic cold spots of intention to use FP services.

Analyzing the 2017 data, as presented in Figure 4b, lower Sindh exhibited an increase in cold spot clusters, indicating a higher prevalence of areas with lower intention to use FP services. This is also the area with the lowest unmet need in Sindh. Only the upper part of Sindh was observed to have high unmet need and cold spots of intention to use. The rest of Sindh, which previously had hotspots of intention to use FP methods later, was not significant in 2017. In Punjab, the situation became more dispersed, with the observation of sporadic hotspot clusters throughout the province indicating a more favorable attitude. The high unmet need became concentrated in the lowest part of Punjab, with few hotspots of intent to use FP services later. No significant changes were observed in KP. Balochistan continued to exhibit cold spots of intention to use FP services later, particularly in areas with high unmet need, indicating a more challenging situation where people do not intend to use FP services despite high unmet need needs.

## 4. Discussion

Family planning efforts in Pakistan have made sluggish progress over the past two decades, failing to achieve desired targets and outcomes. Various factors, including socioeconomic conditions, cultural and gender norms, contraceptive side effects, and the availability and accessibility of services, have been identified as facilitators or barriers to family planning uptake [24–26]. Previous studies have helped profile the users and non-users of contraceptives in Pakistan. However, this research uniquely applied a micro-level lens by harnessing spatial statistical tools to identify high and low uptake clusters, preferred family size, unmet need, and intentions to use FP services in Pakistan. This approach enables the precise identification of “hotspots” and “cold spots” where family planning utilization is significantly higher or lower than national trends. Targeting interventions based on this granular understanding of localization promises to improve efficiency and effectiveness by segmenting patient populations, identifying high-risk communities, and providing engaging visualizations for policymakers and practitioners, akin to successful strategies employed in other public health programs [27,28].

This study revealed substantial geographic disparities and clustering in the uptake of family planning services across Pakistan. The situation of mCPR evolved between 2006 and 2017, with some contraction and diffusion of previously concentrated hotspots. In 2006, mCPR was concentrated in the urban clusters of the provinces. Other researchers who used PDHS 2006–2007 reported that educated women in the upper wealth quintiles living in urban areas were more likely to use family planning services [29]. However, some increase in contraceptive uptake was observed in rural women as well, but primarily because of traditional methods [30]. Sequential analysis of PDHS 2017 indicated an interesting spatial disparity in the distribution of clusters among and within the provinces, especially in Sindh and Punjab. This situation is similar to previously reported findings that showed widespread socioeconomic and regional disparities [31].

Our research findings from hotspot analysis for PDHS 2017 showed that the distribution of mCPR has evolved differently in different geographies. Sindh no longer retained the hotspots of mCPR, indicating a point of concern for program planners. Hackett et al. reported a similar situation, observing a low mCPR in Karachi, an urban center in lower Sindh, despite sufficient client awareness [32]. Simultaneously, the cold spots in the upper Sindh have dispersed, indicating an improvement in mCPR in upper Sindh. This improvement in upper Sindh and the position in lower Sindh demands immediate attention in areas that previously had higher mCPR and no longer retained the same position in 2017.

In contrast, the distribution of the highest CPR for any method and mCPR in Punjab displayed a similar clustering pattern, primarily in and around the urban areas. Balochistan continued to exhibit the lowest prevalence of mCPR in 2017, and the emergence of cold spots throughout the province demands the immediate attention of donors, implementing partners, and government agencies. Sarwar et al. observed a similar pattern of reproductive health services uptake across the country [18].

Our analysis of ideal family size preferences hotspots has unveiled distinct spatial patterns that have demonstrated an inverse association with unmet need in Pakistan. The areas that prefer three or more children were mainly concentrated in regions with moderate to the highest unmet need, indicating a prevailing cultural preference for larger families, which may delay or complicate the adoption of family planning measures. While cultural preference was the strongest predictor of family size and composition [33], in some regions, larger family size was reported as a rational reaction to economic insecurity and persistent conflict [34]. Conversely, metropolitan centers with low unmet need exhibited cold spots for preferences of three or more children, indicating a widespread inclination toward smaller family sizes, usually of fewer than three children. This phenomenon resonates with trends observed in other developing regions, where the unmet need for family planning varies with age and reproductive goals [35]. This prompts a critical question: if individuals with an unmet need for family planning prefer larger family sizes, should they still be categorized as having an unmet need? Our findings underscore the necessity of understanding cultural influence on reproductive goals to comprehend fertility patterns in Pakistan rather than simply addressing supply-side deficiencies.

As revealed in our study, the spatial distribution of unmet need for FP and the intention to use FP services presented intriguing insights into the dynamics of FP preferences across different regions and periods in Pakistan. In 2017, a significant geographical shift was observed in the intention to use in Sindh and Punjab only. Specifically, intention to use was not clustered in areas with high unmet need. The north or upper Sindh, with a significantly higher concentration of unmet need, was resistant to adopting FP services, as observed by the persistent clusters of low intention to use FP.

In Punjab, most upper and central parts exhibited low unmet need and high intention to use FP services, indicating a significant transition from low to high intention to use FP services between 2006–2007 and 2017–2018. Consequently, there was a temporal shift in unmet need across the province, except for the southern or lower Punjab region, which still grappled with high unmet need but displayed significant hotspots of intention to use. Although women in South Punjab intend to use FP services, factors such as limited autonomy in health-seeking behavior and the influence of socio-cultural and religious values act as barriers, preventing young girls and women from accessing family planning [36].

Interestingly, the presence of cold spots of intention to use FP services in areas of high unmet need in upper Sindh, all of Balochistan, and lower KP prompts a reconsideration of the utility of unmet need for FP in policy and program planning. Unmet contraceptive needs contribute to unwanted fertility in low- and middle-income nations, serving as a guide for family planning initiatives. The findings from our study indicated that almost all the areas with the highest unmet need were clustered with cold spots of intention to use FP services in the future. Moreau et al. reported that only half of the women with current unmet need for contraception intended to use contraception in the future, underscoring the complex nature of unmet need, where lack of demand plays a substantial role. The insight emphasized the importance of considering both point prevalence measures of unmet need to understand the current situation and dynamic measures, like unmet demand for contraception to identify women who are interested and willing to use contraception in the future [37].

### 4.1. Methodological Innovation

The present study implemented advanced geospatial techniques, including spatial autocorrelation analysis and hotspot analysis in geographic information system (GIS) mapping, to examine geographic disparities in family planning services uptake in Pakistan. While previous studies have relied primarily on individual or broad provincial or regional comparisons [29,38,39], this research uniquely applied a micro-level lens by harnessing spatial statistical tools to identify clusters of high and low uptake at the more granular level using primary clusters of DHS. The widely used landscape analysis report assessed consumers, service provision, contraceptive supply, and policies and regulations in Pakistan to identify investment opportunities for increasing contraceptive prevalence [3]. Our approach strengthened the existing evidence by precisely identifying “hotspots” and “cold spots” where family planning utilization was significantly higher or lower than national trends, respectively. Interventions that focus on specific “hot and cold spots” based on this granular understanding of localization promise to improve the efficiency and effectiveness of family planning services delivery in Pakistan.

### 4.2. Contribution to the Field

This study makes several notable contributions to family planning and public health research.

1. *Precision Targeted Interventions*: Using geospatial analysis to pinpoint specific geographical areas with the lowest family planning service utilization provides unprecedented detail in identifying vulnerable groups, enhancing service delivery and coverage, and guiding resource allocation and coordinated intervention efforts by policymakers and health organizations. This precision approach allows for developing targeted strategies to address social and behavioral factors, access barriers, and service gaps in the communities with the highest need.
2. *Dynamic Visualization of Disparities*: The geospatial mapping techniques used to generate intuitive visual representations of family planning uptake variations and needs across geographic areas. Compared to traditional statistics-heavy results, these maps enhance comprehension, communication, and decision-making regarding spatial disparities for policymakers, implementation partners, and local communities. The geospatial repositioning of data improves logical inquiries and reasoning, explores complex relationships, and simplifies the conversion and dissemination of information through innovative methods, ultimately making more data-informed decisions.
3. *Temporal Changes and Trends*: The analysis of two-time points identifies temporal shifts in family planning service utilization and changes in geographic hotspots/cold spots. This temporospatial perspective enriches the understanding of evolving dynamics around family planning access, uptake, and continuation and guides public health actions and policy decisions.

### 4.3. Study Limitations

The study had limitations, including reliance on self-reported data from cross-sectional surveys. The exact geographic coordinates of the clusters were displaced to conceal the actual position of the respondents and maintain confidentiality. Urban clusters were displaced up to two kilometers and rural clusters up to five kilometers. However, the researcher ensured that displaced coordinates remained within the actual administrative boundary of the cluster. Hence, the data can identify the geospatial distribution of variables of interest. This study focused on identifying geographic disparities in family planning service uptake through spatial analysis. It did not test associations with factors influencing these spatial patterns. Further qualitative and longitudinal research can provide deeper insights into reasons for clustered disparities between provinces, rural-urban areas, and intention–need gaps.

#### Future Research Directions

This study underscores the necessity for further geospatial analysis to address questions that conventional cognitive approaches have left unresolved. Geospatial methods offer a valuable perspective for understanding and mapping various dimensions of health service provision. Specifically, there is a critical need to map the availability and accessibility of health services, including the presence of trained service providers and essential commodities. Researchers and program planners can gain nuanced insights into demand and supply-side factors hindering FP service uptake.

Moreover, it advocates for collecting geo-data through national and subnational surveys and programs. This approach will facilitate the development of more effective and localized strategies for addressing disparities in FP service availability. It allows program planners to prioritize regions needing enhanced support and resource allocation, ultimately leading to improved service delivery and better health outcomes.

## 5. Conclusions

This study provided valuable insights into the geographic disparities in FP uptake in Pakistan, highlighting the need for targeted interventions to address these variations effectively. By leveraging advanced spatial analysis techniques, including hotspot analysis, this study identified high and low contraceptive prevalence rates (CPR) clusters, offering actionable intelligence for policymakers and healthcare practitioners. These findings highlighted the importance of tailoring interventions to the specific geographic areas identified through hotspot analysis, with the potential to optimize resource allocation and improve FP service delivery, particularly in marginalized and underserved areas. Furthermore, identifying geographic disparities in FP uptake should stimulate meaningful stakeholder collaborations to drive policy reform. The complex challenges limiting FP uptake in Pakistan can be most effectively addressed through prioritized, evidence-based decision-making and investments.

## Figures and Tables

**Table 1.** Spatial Autocorrelation by Moran Index.

Indicators	PDHS 2006–2007		PDHS 2017–2018	
	Global Moran Index	*p*-Value	Global Moran Index	*p*-Value
CPR Any Method	0.427	<0.001	0.483	<0.001
CPR Modern Methods	0.339	<0.001	0.304	<0.001
Unmet Need	0.077	<0.001	0.165	0.002

## Data Availability

The data used for this study was obtained from the Pakistan Demographic and Health Surveys (PDHS) that were collected between 2006–2007 and 2017–2018. The PDHS is a nationally representative household survey conducted every five years. Details on the sampling strategy can be accessed at the DHS Program website (“Sampling and Household Listing Manual [DHSM4]”). Data is available with the DHS Program of ICF at https://dhsprogram.com/data/available-datasets.cfm and can be accessed upon approval of a formal request to the DHS Program.
